# Success in the Natural Detection Task is influenced by only a few factors generally believed to affect dogs’ olfactory performance

**DOI:** 10.1038/s41598-024-62957-5

**Published:** 2024-05-29

**Authors:** Attila Salamon, Eszter Baranya, László Róbert Zsiros, Ádám Miklósi, Melitta Csepregi, Enikő Kubinyi, Attila Andics, Márta Gácsi

**Affiliations:** 1 HUN-REN-ELTE Comparative Ethology Research Group, Budapest, Hungary; 2 ELTE NAP Canine Brain Research Group, Budapest, Hungary; 3 Department of Ethology, ELTE Eötvös Loránd University, Budapest, Hungary; 4 Hungarian Ethology Foundation, Göd, Hungary; 5 Doctoral School of Biology, Institute of Biology, ELTE Eötvös Loránd University, Budapest, Hungary; 6 MTA-ELTE Lendület “Momentum” Companion Animal Research Group, Budapest, Hungary; 7 Neuroethology of Communication Lab, Department of Ethology, Eötvös Loránd University, Budapest, Hungary

**Keywords:** Dog, Olfaction, Natural Detection Task, Age, Test location, Bayesian statistics, Animal behaviour, Olfactory system

## Abstract

Research into dogs' olfactory ability is growing rapidly. However, generalising based on scientific results is challenging, because research has been typically conducted on a few specially trained subjects of a few breeds tested in different environmental conditions. We investigated the effects of temperature and humidity (outdoors), age, test location, sex, neutering status, and repeated testing (outdoors and indoors) on the olfactory performance of untrained family dogs (N = 411) of various breeds. We employed the Natural Detection Task with three difficulty levels, from which we derived two performance metrics: Top Level and Success Score. Temperature (0–25 °C) and humidity (18–90%) did not affect olfactory performance. Young adult dogs surpassed other age groups in reaching the Top Level. Sex and neutering status showed no discernible influence on Top Level and Success Score. Dogs performed better in both metrics when tested indoors compared to outdoors. In the test–retest procedure no significant learning effect was observed. We confirmed on untrained companion dogs that olfactory performance declines with age and rejected some factors that have been previously hypothesised to significantly affect dogs’ olfactory success. The influence of the testing environment was notable, emphasising the need to consider various factors in understanding dogs' olfactory capabilities.

## Introduction

Although numerous technical devices have been invented and being developed for the detection and discrimination of volatile odours (e.g., 3D printed dog’s nose^1^; near-infrared optical nose—NIRON^2^; bio-electronic nose—BEN^3^; for a review see^4^), nowadays dog’s olfactory ability is still used in a wide range of tasks and the related research is expanding. Dogs have been used in numerous fields with great success: in the military and police forces for explosive and drug detection^5,6^, in customs to find banknotes and tobacco^7^, in the medical field for disease detection^8,9^, in human scent detection^10^, for search and rescue^11^, and in hunting and conservation to detect scats, carcasses or live animals^11,12^.

Most previous studies attempted to measure dogs’ olfactory ability by using a small number of dogs trained to identify biologically irrelevant scents (e.g., black tea^13^; explosives^14^). Since detection dogs have many specific characteristics^15,16^, the results of research focusing only on them do not give a complete picture of the olfactory abilities of dogs in general, and ignore breeds such as bloodhounds, which are considered to have the best sense of smell, but show little controllability and motivation^17^. Thus, Polgár et al.^18^ developed the Natural Detection Task (NDT), a simple procedure in which dogs have to find a certain amount of food in a natural search situation. The NDT is a standard olfactory test that enables the testing of untrained family dogs and allows for the examination of several factors that would otherwise likely be ruled out when using trained dogs (e.g., personality). In the NDT, the relative differences in olfactory sensitivity can also be quantified by applying increasingly difficult levels. Polgár et al.^18^ compared scent breeds (dogs bred specifically for the purpose of performing scent-related jobs), non-scent breeds (dog breeds which have been selected for jobs that require characteristics not related to olfaction) and short-nosed (brachycephalic) breeds as well as captive, hand raised and socialised grey wolves (*Canis lupus*). They only found a difference in the hardest level (target hidden in an airtight plastic container with no holes on its lid), with only scent breeds and wolves performing better than the chance level.

The NDT was originally developed as an outdoor field test because of the wolves^18^, and naturally, different environmental factors can influence dogs’ olfactory performance outdoors, for example, temperature and humidity (see^19^), though some studies did not find such effects (e.g.^20,21^). In lower temperatures, the bacterial activity is lower, which can negatively affect the amount and spread of odour emitted from organic samples^22^. In previous studies, higher temperatures negatively affected dogs’ search performance^23^ and their olfactory abilities due to more frequent panting^14^ and due to the risk of dehydration resulting in decreased enzyme activity and nasal mucosal fluidity^24^. Wohlfahrt et al.^25^ found that olfactory sensitivity was not influenced by temperature below 30 °C and humidity higher than 50%, but decreased olfactory specificity was observed at higher temperatures (30–35 °C) and lower relative humidities. Other studies also showed that increased humidity positively influenced tracking efficiency^26,27^, and improved nasal humidity and enhanced odorant trapping^24^.

In addition to weather conditions, other factors may affect how dogs’ olfactory performance differs in a field versus an indoor setting. Outdoors, there is a wide range of distracting stimuli, such as scents, moving objects, and animals, which are necessary to simulate real-life situations for detection dogs. In contrast, testing indoors allows for the control of environmental factors, such as temperature or air flow that may affect the dispersion of scents, as well as the elimination of visual and olfactory stimuli^18^.

So far, only a few studies have compared the olfactory performance of detection dogs in indoor and outdoor (more life-like) environments. Gazit and Terkel^14^ tested 6 explosive detection dogs indoors and outdoors and found that all dogs detected the target (30 g of C4 explosive) indoors, while detection success was 91% outdoors. Jezierski et al.^5^ tested trained drug detection dogs to find 10–15 g of marijuana, amphetamine, hashish, heroin, or cocaine, which were hidden in unsealed plastic bags 1 h before searching and found no difference in the percentage of correct indications between indoors and outdoors. Interestingly, Abdel Fattah and Gharib^28^ tested trained drug detection dogs using similar procedures to Jezierski et al.^5^ and found that detection dogs performed better indoors compared to outdoors.

Age-related decline in olfactory function in dogs has not been reported in as much detail as in humans^29^. A few studies reported atrophic changes in the olfactory epithelium and a decrease in the number of olfactory and supporting cells in aged dogs^30^, possibly due to reduced regenerative capacity and increased apoptosis^29^. Further, the olfactory bulbs shrink with age^31^ and contain senile brain lesions in aged dogs^30^. These changes likely decrease the olfactory ability of dogs. Based on the owners’ perception, age-related decline in dogs’ olfactory ability was reported^32^, although many studies have not found any age effect in olfactory tasks (companion dogs^33–36^; working dogs^7,37^), which might be explained by the different age range of the samples.

Sex did not affect olfactory performance in most studies (companion dogs^33–35^; working dogs^7,38^). However, in the case of narcotic detection police dogs, males and intact dogs were reported to perform better in detecting the target material than females and neutered ones^39^.

Sex and neutering status may have little direct relevance in olfactory tests, yet it is important to note that each sex may have characteristics that may become important in a detection task. For example, females are generally easier to control^40^, and female Labradors scored higher for the ability to cooperate than males in various test situations, though the opposite was found in German shepherds: males scored higher for the ability to cooperate compared to females^15,41^. However, there was no sex difference in a large sample of dogs in their tendency to rely on the human pointing gesture^42^. Neutering has been reported to decrease distractibility^15,43^, increase trainability^44^, and neutered dogs scored higher for ‘desire to work’^43^. However, racing greyhounds are sometimes not neutered or spayed to maintain their high energy level^45^.

In this study, testing a very large sample of untrained companion dogs, we focused on the above factors that were thought to be important from a practical point of view, but so far have only been examined on small samples and most studies did not yield generalisable results.

First, we examined whether temperature and humidity have an effect on the dogs’ performance in the NDT outdoors^18^. We expected that dogs would either perform worse at lower and higher temperatures, or the potential influencing effects would not prevail within the temperature range we applied (0–25 °C). Further, we predicted that dogs will perform better in higher humidity.

Second, we compared dogs’ performance outdoors and indoors in the NDT. We assumed that dogs would either perform better indoors due to fewer visual and/or olfactory disturbances^18^, or there would be no difference, as the accumulation of the target odour in the test room could also distract the dog.

Then, we examined the potential effects of dogs’ age, sex and neutering status on their success. We assumed that there would be either no age effect on the olfactory performance consistent with some previous studies (e.g., companion dogs^33^; working dogs^7^), or there would be a decline in the case of older dogs’ due to the physiological changes occurring in aged dogs’ olfactory system^30^ or the deterioration of aged dogs’ cognitive abilities (e.g., concentration). For the effect of sex and neutering status, we only examined dogs older than 1.5 years of age, because juvenile dogs are more likely to be intact. Similarly to most previous studies, we did not expect sex differences. Regarding the neutering status, we assumed that it either would not affect olfactory performance or would increase olfactory performance due to the positive effects on some traits, such as distractibility and desire to work (e.g.,^43^).

## Methods

### Ethics statement

The ethical approval for this study was granted by the Animal Welfare Committee of Eötvös Loránd University (ELTE-AWC-020/2018 and ELTE-AWC-015/2023). All methods were carried out in accordance with relevant guidelines (including ARRIVE) and regulations, the experiment was performed in accordance with the EU Directive 2010/63/EU and the recommendations of the Hungarian State Health and Medical Service. All owners with their dogs were recruited through social media and from the Family Dog Project database. An informed consent was obtained from all dog owners and they participated in the test with their pets on a voluntary basis. Owners were told that they could terminate the experiment at any time when they thought their pet was being exposed to unwanted stress.

### Subjects

In this study, we tested the olfactory performance of 434 dogs (> 6 months old) in one of two locations; outdoors or indoors. From these, 23 dogs were excluded (11 outdoors and 12 indoors) because they did not even pass Level 1, which was planned to serve as a positive control. Apparently, these dogs either did not understand the task situation or were not motivated enough to search for the food (even though they passed the motivation test).

To assess the effects of age and test location, we evaluated the data of 149 dogs tested outdoors, comprising 12 breeds and 4 mongrels. This group included 73 males (35 neutered) and 76 females (53 neutered), with ages spanning from 0.5 to 16 years (mean: 5.22, SD: 4 years). We also analysed data from 262 dogs tested indoors, encompassing 13 breeds and 5 mongrels. This set had 129 males (72 neutered) and 133 females (93 neutered), with ages ranging from 0.5 to 15 years (mean: 3.9, SD: 3.4 years). The outdoor and indoor groups were balanced for sex, age groups and neutering status as much as possible.

To assess the effects of sex and neutering status, we only considered dogs > 1.5 years of age and balanced the groups for test location, and age groups. We evaluated the data of 120 dogs tested, comprising 13 breeds and 3 mongrels. This group included 60 males (30 neutered) and 60 females (30 neutered), with ages spanning from 2 to 10 years (mean: 4.26, SD: 2.08 years).

We assessed the effect of test location and test order in a within-subject design. Of the 57 retested dogs, 27 began their tests outdoors and 30 indoors; these were balanced for age groups as much as possible. Representing 11 breeds, the group comprised 33 males (18 neutered) and 24 females (19 neutered). Their ages on the first occasion varied from 0.5 to 12 years (mean: 4.02, SD: 3.33 years). The retests were conducted on a separate occasion, on average 3 months after the first test.

### Location

**Figure 1:**
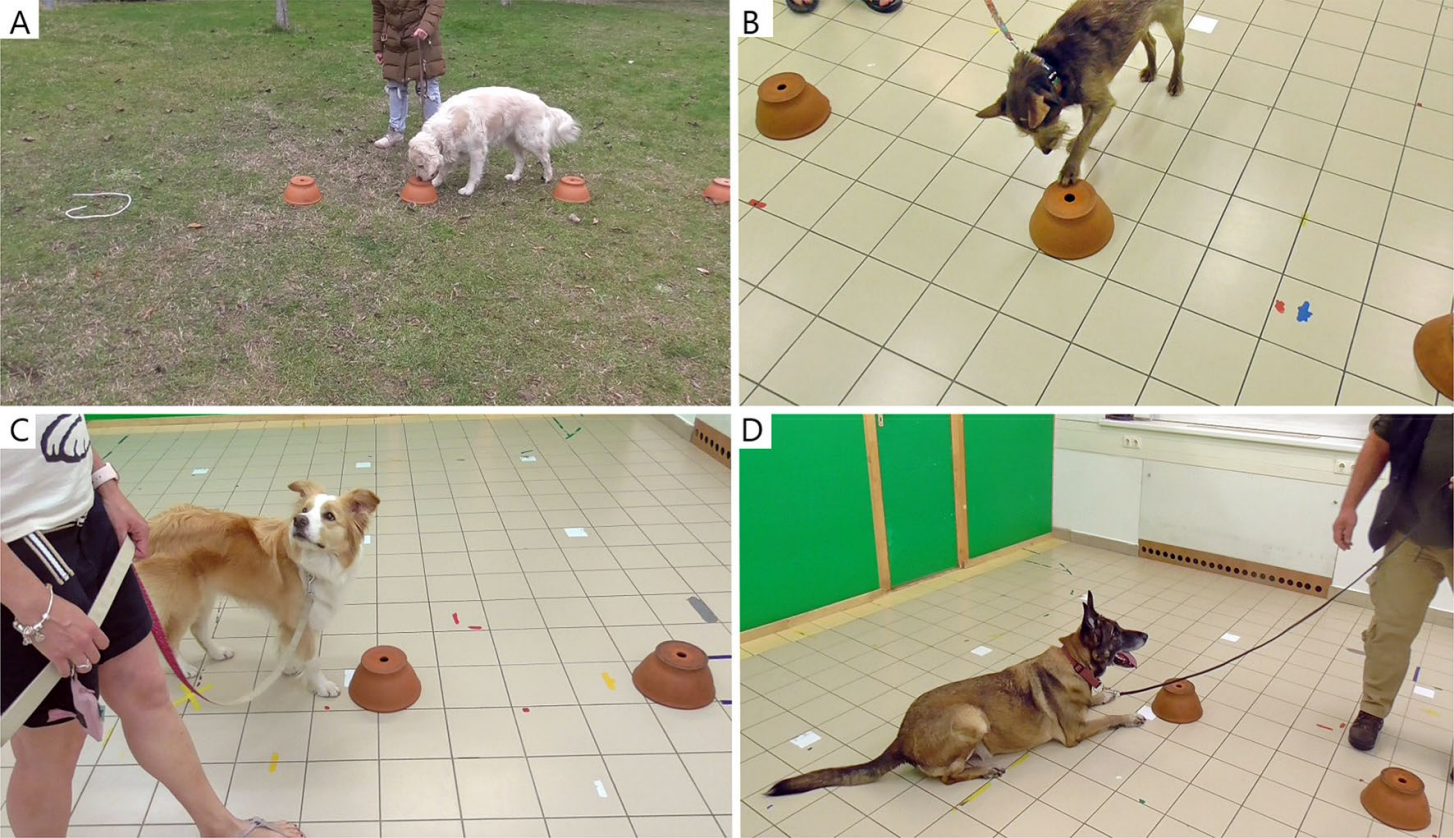
Test locations and indications. (**A**) Outdoor test location; flat grassy area surrounded by hedges and trees, relatively isolated from pedestrians and dog walkers. (**B–D**) Indoor test location; clean and empty laboratory room. Dogs’ spontaneous indications of the target scent (4 typical types): (**A**) nose in the hole for > 2 s, (**B**) pawing, (**C**) looking at the owner, (**D**) lying down.

The tests were conducted in one outdoor and one indoor location. The outdoor area (see Fig. 1A) was a flat grassy area surrounded by hedges, thus relatively visually isolated from potential distractions. This area was not frequented by dog walkers, minimising the amount of distracting scents or visual effects. The indoor location was a 6.3 × 5.4 m test room at the Department of Ethology, Eötvös Loránd University (see Fig. 1B–D). The test room was continuously ventilated and thoroughly cleaned after each test.

### Temperature and humidity

The outdoor temperature and humidity data for each test was collected by the meteorological station of Eötvös Loránd University situated approximately 50 m from the outdoor test location. The hourly meteorological data was freely available from the Meteorological Database of the Hungarian Meteorological Service. The average temperature was 13.98 ± 6.12 °C (± SD, range: 0–25 °C), and the average humidity was 53 ± 19.37% (± SD, range: 18–90%).

The temperature in the test room was set at 22 °C that could vary ± 1 °C with humidity ranging between 45 and 75%.

### Procedure

The test procedure was a modified version of the NDT (see^18^); in addition to some changes in the evaluation methods (see below in detail), we used three levels instead of five, as suggested by the authors in their discussion.

To ensure that dogs were motivated to search for food the owners were instructed not to feed their dogs in the 3 h preceding the test. Right before the tests began, the dog’s motivation was checked by offering a piece of bait from an open container placed on the ground in front of the dog; one dog could not be tested based on this criteria. The test could only be started if the dog ate the bait from the container. The hypoallergenic Alpha Spirit Only Fish semi moist food was used as bait exclusively.

During the test, four round ceramic pots (23 cm diameter), each with a 2.5 cm hole on the bottom, were placed upside-down in a straight line at approximately one-metre intervals. Starting lines were placed 1.5 m away from each end of the pot line.

There was a plastic container (Curver brand, 0.25 l) with a lid under each pot. Three of the containers were “no food” containers, and never came into contact with any food or any persons who handled the food. A number of other containers were used for the baiting so that they could be regularly changed.

Two experimenters (E) were needed to run the test. E1 set up the pots and placed the baited container in the predetermined position. He/she used a teaspoon to place the bait into the container and never touched it by hand. E2 never came into contact with the bait, pots or containers. He/she was also blind to the location of the baited container. E2 explained the test procedure to the owner and was filming the trials using a handheld camera (Panasonic HC-V180) or a smartphone. The owner and the dog did not view the baiting of the pots and faced away from the test area until they were called. In the case of indoor tests, the owner and the dog left the test room for the time of baiting.

Once the bait was placed under a pot, E2 started a stopwatch. When E2 placed the food in the container under the pot, its scent began accumulating (first in the container, then in the pot). We waited one minute because based on the methods of Polgár et al.^18^ and in the pilot tests we ascertained that this time was necessary for the odour to flow out to a sufficient extent. After one minute, E1 summoned the owner and the animal to the starting line, allowing them to begin walking the path on a loose leash. Before starting, the owner could provide an encouraging command (such as "find it!”, or "search!”), but no communication or gestures were allowed while walking or when the dog examined the pots. The only exception was calling the dog back with an encouraging tone if it moved away from the setup, saying, "come here, where’s the food?" (see^18^).

The animal could smell each pot, while walking along the setup. The trial ended either when the animal indicated one of the pots or when one minute of active searching elapsed without indication. An indication was defined as any of the following behaviours: refusing to move away from pot; attempting to turn the pot over; placing paw on pot; poking or pushing the pot with the nose; vocalising while next to pot; significantly increasing tail wagging speed while sniffing pot; sitting or lying down next to the pot (see Fig. 1A–D). The dogs were not taught any indication, they spontaneously indicated the food bait under the pot. The indication, determined by E2, was only counted if lasting more than 2 s. If the indication lasted less than 2 s and the dog moved on, it was allowed to continue searching. If the dog turned the pot over, it was an indication, and the owner had to grab the container at once without waiting for E2. If the owner and the animal reached the end of the line without making an indication, they were permitted to turn back around (when the dog could be encouraged again) and walk the line from the opposite direction until the one minute elapsed. After each trial, there was a 1–2-min waiting period when the pots were set up for the next trial. During this time, the dog remained on the leash and was allowed to drink and rest (see^18^).

The results of each trial could be classified into one of four categories: correct choice, incorrect choice, no choice, and no attempt.

Correct choice: the choice was correct if the animal indicated the baited pot within the allowed time. In this case, the subject was allowed to eat the bait from the container. The owner lifted the pot up, opened the container, and allowed the dog to eat the bait. The owner was not permitted to touch the bait by hand.

Incorrect choice: the choice was incorrect if the animal indicated an unbaited pot. In this case, the owner showed the dog that the container under the indicated pot contained no food.

No choice and no attempt: we recorded no choice if the subject sniffed the pots but did not make an indication within one minute of active searching. The trial was recorded as no attempt if the subject did not examine all the pots or showed interest in them or if the subject repeatedly moved away from the row. If there were three consecutive no-choices and/or no attempts, the test was ended. If the dog did not choose (no choice or no attempt), then it was not shown any of the pots.

The baited container, the lid and the pot were replaced with clean ones for the next trial. They were aerated for one trial and then reused as a non-baited container (the incorrectly indicated pot and container were not replaced).

Based on experiences from the pilot studies, and in order to facilitate rapid learning of the task, we chose a procedure that uses progressively more difficult levels. As suggested by Polgár et al.^18^, we used three levels of difficulty determined by the type of lid placed on the baited container.

- In Level 1, the containers had an open top with no lid. As Level 1 was not an olfactory challenge (see^18^), this level only served as a pre-test and controlled for (a) understanding the setup/task, (b) motivation in a task situation (to act independently), and (c) the use of alternative problem-solving methods instead of using olfaction (such as turning all pots upside down). Therefore, the data of dogs failing this level was not analysed.
- In Level 2, the containers’ lids had five holes (each 0.5 cm in diameter) drilled into them.
- In Level 3, the container was covered completely with a regular lid (no holes).

The subject passed to the next level, if it had at least 3 correct choices from the last 4 trials. If a subject could not pass a level within 12 trials, the test ended. Thus, there were a minimum of 3 and a maximum of 12 trials at each level (a total of 3–36 trials), where the baited container was under each of the four pots in a random order. The bait was never under the same pot twice consecutively.

In order to decrease the likelihood that the animals would use cognitive strategies or local enhancement to indicate at a pot^46^, after each trial, the row of pots was rotated to a different angle and shifted over at least 1 m by E1 on the field or in the test room. The starting line was also shifted to the opposite side for each trial.

### Data pre-processing

Data pre-processing and cleaning were performed using Python (v. 3.11.3; McKinney et al.^47^) with the pandas library (v. 2.0.1; The pandas development team^48^).

**Figure 2:**
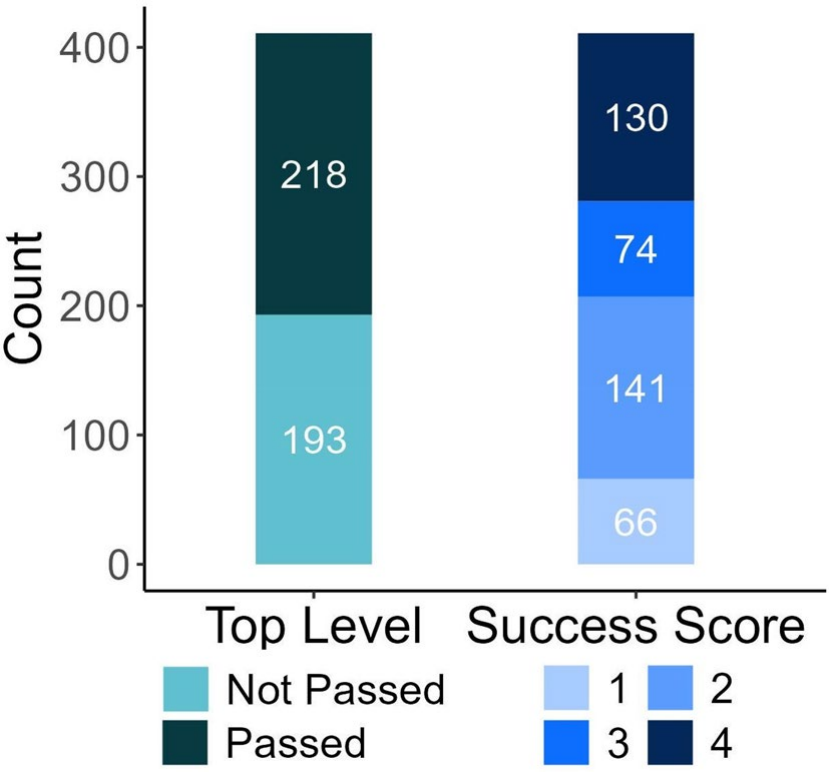
Illustration of the two metrics, Top Level and Success Score, used to characterise the performance of dogs (N = 411) in the olfactory test. The number of dogs are shown for each category within the two metrics.

We utilised two main metrics to evaluate the olfactory performance of dogs: Top Level (TL) and Success Score (SUS) (see Supplementary Information). TL, a Boolean variable, simply indicates whether a dog managed to pass the third and most challenging level of the NDT. On the other hand, SUS is a multifaceted, ordinal metric that integrates the levels a dog passed and the number of trials taken to achieve those levels. We made up two categories based on the number of trials, as the data naturally distributed into two groups; dogs that made no or one mistake (succeeded within 3–4 trials) and other dogs that made more mistakes. The SUS metric was developed in a way to be more sensitive to dogs’ performance compared to TL, but still have a large enough sample size in each of its categories. The specific calculation for the Success Score can be found in Table 1, while the illustration of these two metrics used to characterise the subjects’ performance in the NDT is shown in Fig. 2.

**Table 1:** Measured variables related to the performance and the calculation of the Success Score (SUS).

Passed level	Number of trials (level 2)	Number of trials (level 3)	Top level	Success score
1	–	–	0	1
2	5–12	–	0	1
2	3–4	–	0	2
3	5–12	5–12	1	2
3	3–4	5–12	1	3
3	5–12	3–4	1	3
3	3–4	3–4	1	4

The subjects had a maximum of 12 trials on each level and they passed a level, if they had at least 3 correct choices from the last 4 trials.

**Figure 3:**
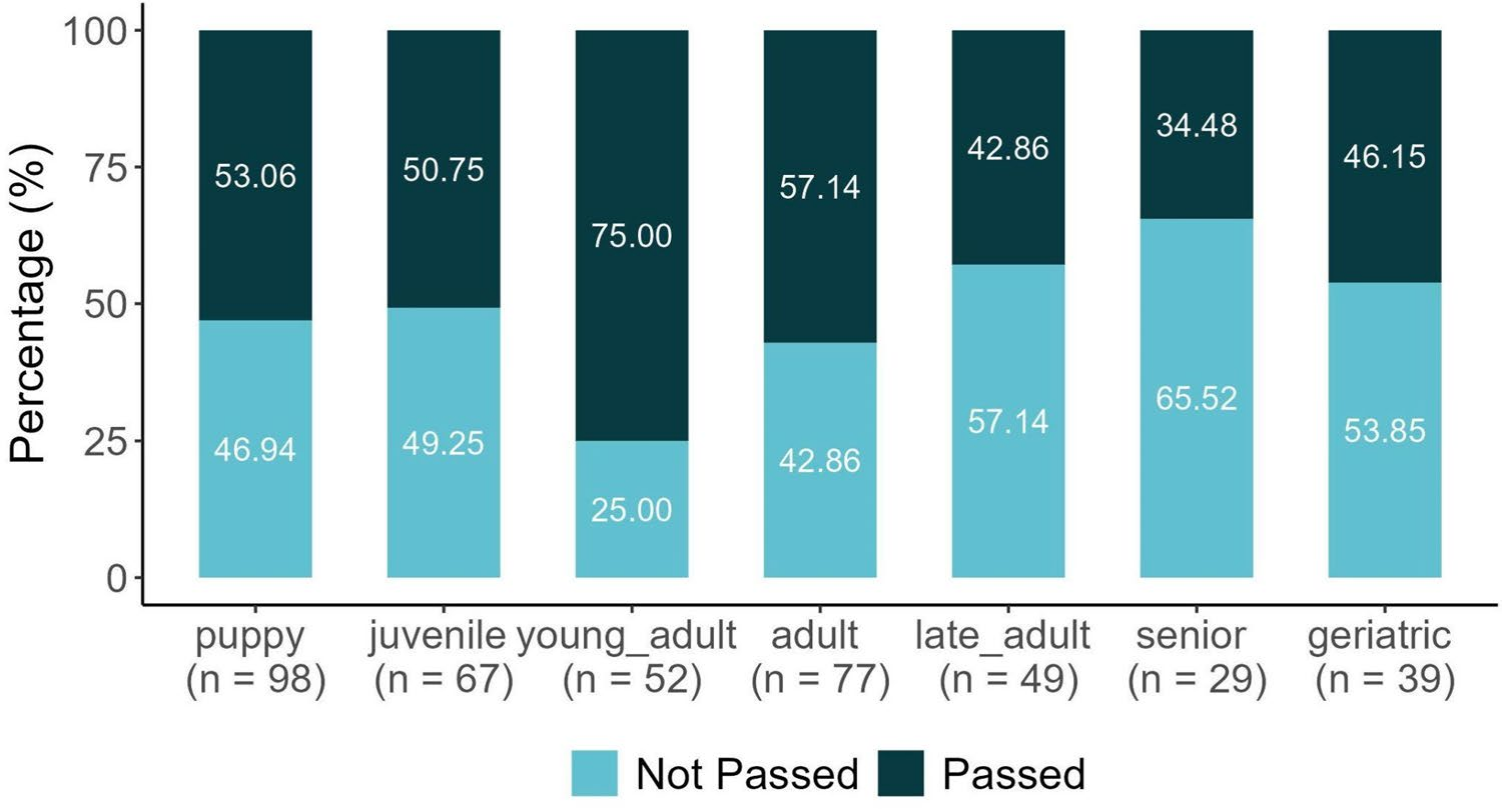
The percentage of dogs reaching Top Level in each age category (total N = 411).

Although using age data as a continuous variable has several advantages, we formed categories for the following reasons. In the questionnaire the owners gave the dogs’ age in months up to 1 years of age, and in years after 1 years of age, because the exact birth date of the dogs was not always known. Further, we expected a non-linear relationship between age and olfactory performance. We categorised dogs' ages into seven distinct age groups (see Fig. 3): 'puppy' (< 1 year), 'juvenile' (1–2 years), 'young adult' (2–3 years), 'adult' (3–6 years), 'late adult' (6–8 years), 'senior' (8–10 years), and 'geriatric' (> 10 years), based on classifications used by previous studies (e.g., see^49,50^).

### Statistical analysis

All statistical analyses were conducted in R (v. 4.2.2; R Development Core Team^51^) using RStudio (v. 2022.7.1.554; RStudio Team^52^). Basic statistical analyses were performed using native R statistical functions. The tidyverse suite of packages facilitated data manipulation and transformation as well as visualisation^53^.

The effects of temperature and humidity on the two response variables were analysed on the outdoor sample employing Kruskal–Wallis tests.

To investigate the potential effects of age and test location on the combined outdoor and indoor sample, we initially used traditional hierarchical frequentist models. However, we found these approaches to be insufficiently robust in terms of explanatory power. To address these limitations, we turned to Bayesian generalized linear regression models (GLM). Bayesian methods supported our aim, as they can achieve a more nuanced understanding of the underlying distributions and effects, especially given the complex nature of our data.

As there was a moderate association between the age and the neutering status (Cramér’s V = 0.45, p < 0.001), a separate model on a subsample balanced for location and age group was necessary to examine the effects of sex and neutering status. Thus, a second Bayesian GLM (for both metrics) incorporated sex, neutering status and their potential interaction. For the within-subjects design (retested dogs), Bayesian generalized linear mixed models (GLMM) were used for both variables. These models accounted for test location, test order, their potential interaction, and the dogs’ ID as the random effect.

Bayesian analyses were carried out using the brm function from the brms package^54^, specifically employing cumulative link models for ordinal outcomes (SUS) and Bernoulli models for boolean outcomes (TL). We also assessed the adjacent category model, which did not enhance model fit, affirming our model choice. In the Monte Carlo Markov Chain process there were 2000 iterations, running four chains in parallel to ensure precise estimation. For each of our Bayesian models we utilized weakly informed priors (normal distribution centred at 0 with a standard deviation of 2.5) after confirming through a sensitivity analysis that these priors provided a robust fit to our dataset. The sensitivity analysis was carried out by using at least three sets of priors of varying width for each model. Robustness was evidenced by fairly low differences in estimates, at least moderately high bulk effective sample sizes (ranging from approximately 1200 to 4800), and Gelman-Rubin convergence statistics (R-hat values) consistently at 1, indicating reliable parameter estimates. All our Bayesian GLMs were selected based on thorough consideration by evaluating commonly used frequentist (e.g., Akaike Information Criterion, adjusted R^2^) and Bayesian performance metrics (e.g., Leave One Out Cross-Validation, Watanabe-Akaike Information Criterion), as well as by visually inspecting graphical diagnostic plots (Posterior Predictive Check).

## Results

### Temperature and humidity

The effect of temperature and humidity were examined only on dogs tested outdoors (N = 149). No significant association was revealed between temperature and either TL (χ^2^ = 0.01, p = 0.921) or SUS (χ^2^ = 5.26, p = 0.154) within the range of 0 and 25 ºC. Similarly, humidity did not show a significant association with TL (χ^2^ = 0.53, p = 0.467) or SUS (χ^2^ = 4.89, p = 0.18).

### Age

Young adults reached TL in the highest percentage (Fig. 3); thus we used them as the reference category for the statistical analysis. All other age categories decreased the odds of reaching TL compared to the reference category, young adults (see Table 2): puppies (62%), juveniles (66%), adults (50%), late adults (72%), seniors (81%), and geriatrics (66%). However, the upper edge of the Credibility Interval (CI) for the adult category did encompass 1, suggesting slight uncertainty in this result (Table 2). Especially dogs in the older age categories seem to show significantly poorer performance (Fig. 3).

**Table 2:** Odds ratios and the lower and upper 95% of their credibility intervals (CI) for the factors (age and test location) examined in relation to the Top Level and Success Score variables.

	Top level			Success score		
	Odds ratio	L-95% CI	U-95% CI	Odds ratio	L-95% CI	U-95% CI
Intercept 1	3.5	1.88	6.63	0.09	0.05	0.16
Intercept 2	–	–	–	0.53	0.32	0.87
Intercept 3	–	–	–	1.16	0.7	1.9
Puppy (age)	0.38	0.18	0.78	0.62	0.35	1.1
Juvenile (age)	0.34	0.16	0.73	0.6	0.32	1.13
Adult (age)	0.5	0.22	1.05	0.66	0.35	1.19
Late adult (age)	0.28	0.12	0.63	0.36	0.18	0.71
Senior (age)	0.19	0.07	0.47	0.37	0.17	0.82
Geriatric (age)	0.34	0.14	0.8	0.61	0.3	1.24
Outdoors (location)	0.56	0.36	0.86	0.67	0.46	0.98

The effect of a factor can be considered significant if 1 does not fall between the L-95% CI and U-95% CI values. The uncertainty of the effect of a factor increases with the width of the CI range, and the closer the centre of the range is to 1.

Puppies, juveniles, adults and geriatric dogs showed reductions in the odds of achieving a higher SUS compared to young adults as the reference category, with decreases of 38%, 40%, 34%, and 39%, respectively. However, the CIs for these categories all touch upon 1, hinting at potential uncertainty in these results. Late adults and seniors, however, had a more definitive decrease in the odds with 64% and 63%, respectively. The CIs for both these categories did not contain 1, reinforcing the statistical significance of these results (Table 2).

### Test location

**Figure 4:**
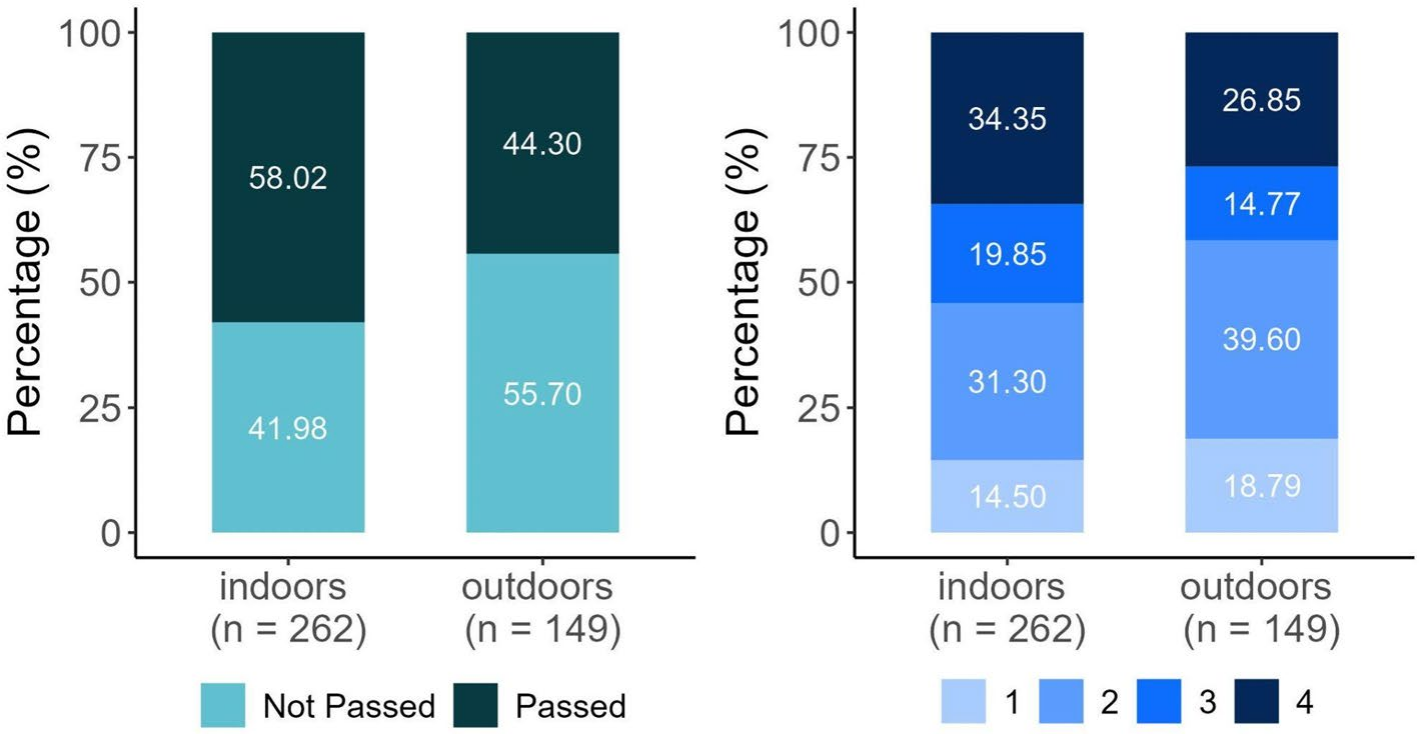
Top Level and Success Score results of dogs in the two test locations (total N = 411).

As for the effect of the test location, a higher proportion of dogs reached the TL indoors (Fig. 4). Tests conducted outdoors were associated with a 44% decrease in the odds of a successful outcome compared to those conducted indoors. The absence of 1 within the CIs confirmed the statistical significance of this result (Table 2).

A higher percentage of dogs achieved greater SUS indoors compared to outdoors (Fig. 4). Tests conducted outdoors were associated with a 33% decrease in the odds of a higher SUS compared to those conducted indoors. The absence of 1 within the CIs confirmed the statistical significance of this result (Table 2).

### Sex and neutering status

**Figure 5:**
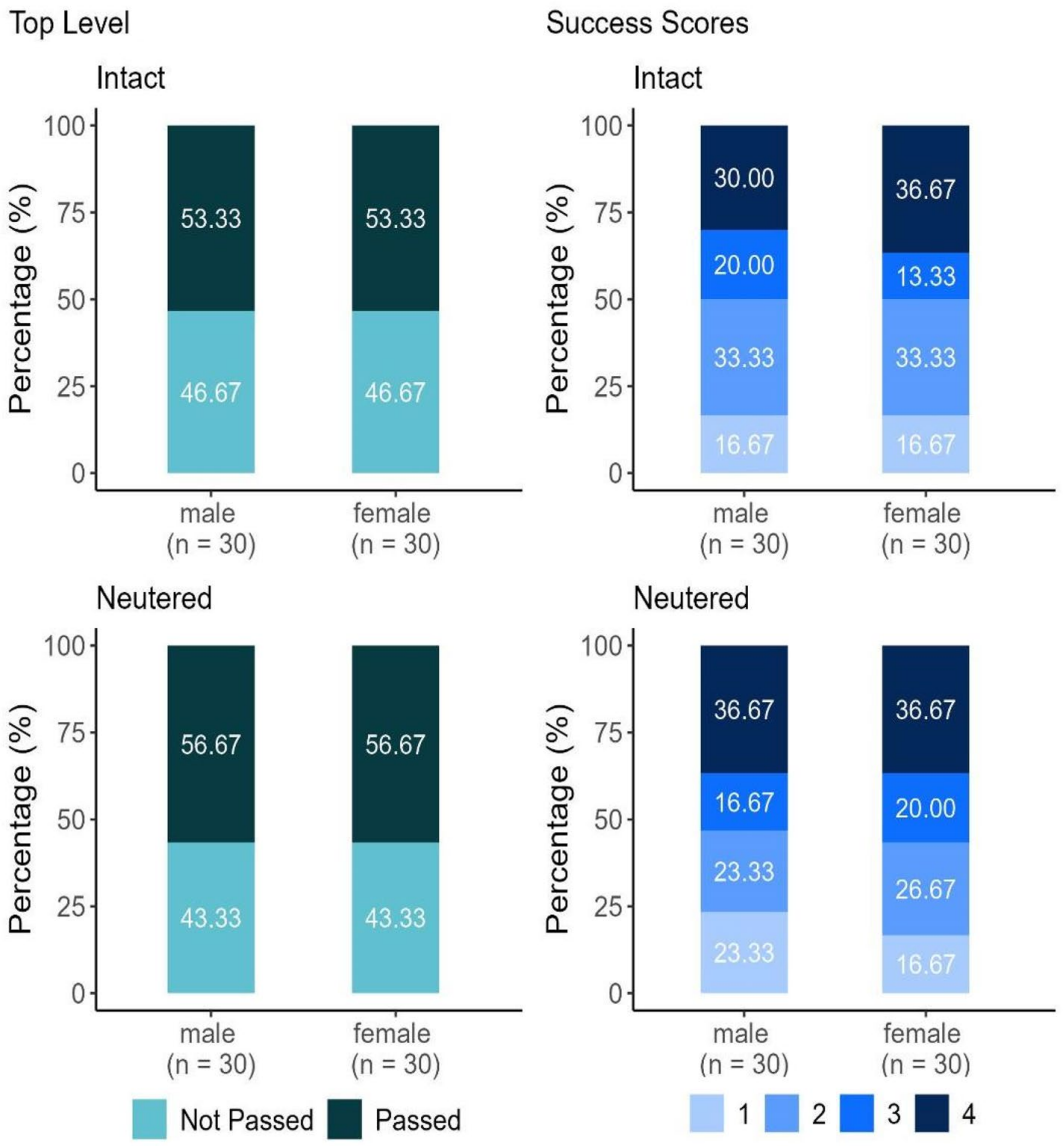
Top Level and Success Score results of dogs over 2 years of age, depending on their sex and neutering status (N = 120).

There was no effect of sex, because the CI has a wide range and the position of 1 within the CI introduces a high level of uncertainty (Fig. 5). Neutered dogs had a 12% increase in the odds of the successful outcome compared to intact ones. The wide range of the CI and the position of the 1 within the CI suggest no significant effect of the neutering status. In terms of sex–neutering interaction (using the performance of neutered females as a reference), no significant effects were found (Table 3).

**Table 3:** Odds ratios and the lower and upper 95% of their credibility intervals (CI) for the factors (sex and neutering status) examined in relation to the Top Level and Success Score variables in both locations.

	Top level			Success score		
	Odds ratio	L-95% CI	U-95% CI	Odds ratio	L-95% CI	U-95% CI
Intercept 1	1.17	0.58	2.32	0.24	0.12	0.47
Intercept 2	–	–	–	0.99	0.52	1.88
Intercept 3	–	–	–	2.09	1.08	4.06
Female (sex)	0.98	0.38	2.65	1.12	0.46	2.7
Neutered (neutering status)	1.12	0.4	3.02	1.05	0.42	2.6
Female × neutered	1.03	0.27	4.08	1.04	0.31	3.64

The effect of a factor can be considered significant if 1 does not fall between the L-95% CI and U-95% CI values. The uncertainty of the effect of a factor increases with the width of the CI range, and the closer the centre of the range is to 1.

Females showed a 12% increase in the odds of a higher SUS compared to males. The CI has a wide range, and the position of 1 within the CI introduces a high level of uncertainty, which suggests no significant sex effect (Fig. 5). There is no effect of neutering status due to the wide range of the CI and the position of the 1 within the CI. In terms of sex–neutering interaction (using the performance of neutered females as a reference), no significant effects were found (Table 3).

### Test–retest comparison

**Figure 6:**
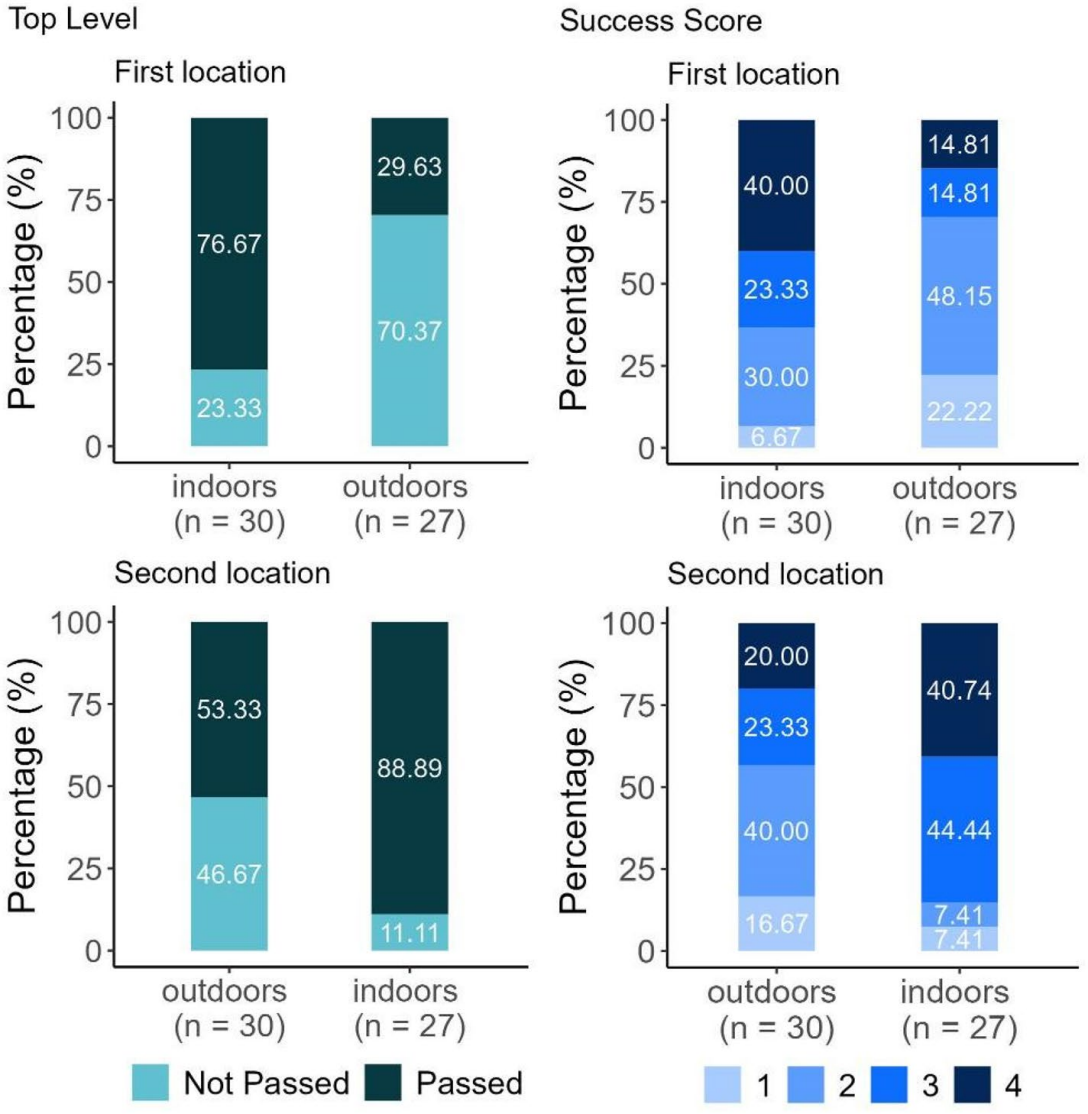
The Top Level and Success Score results of dogs in the two test locations and test orders (within-subject design) (total N = 57).

We found that in the within-subject design there was a 96% decrease in the odds of reaching the TL outdoors compared to indoors, with a high certainty due to the absence of 1 in the CI. No order effect was detected in reaching TL due to the wide range of the CI that encompassed 1 (Table 4, Fig. 6). In terms of the test location–test order interaction (using the performance outdoors location and second test as reference), no significant effects were found (Table 4).

**Table 4:** Odds ratios and the lower and upper 95% of their credibility intervals (CI) for the factors (test location and test order) examined in relation to the Top Level and Success Score variables in the test–retest comparison.

	Top level			Success score		
	Odds ratio	L-95% CI	U-95% CI	Odds ratio	L-95% CI	U-95% CI
Intercept 1	6.17	1.8	28.44	0.03	0.007	0.1
Intercept 2	–	–	–	0.39	0.15	0.99
Intercept 3	–	–	–	2.32	0.95	6.25
Outdoors (location)	0.04	0.005	0.24	0.16	0.04	0.52
Repeated test (order)	4.56	0.75	39.19	1.78	0.55	6.09
Outdoors × repeated test	0.95	0.07	11.79	1.04	0.16	6.65

The effect of a factor can be considered significant if 1 does not fall between the L-95% CI and U-95% CI values. The uncertainty of the effect of a factor increases with the width of the CI range, and the closer the centre of the range is to 1.

The outdoor location decreased the odds of reaching a higher SUS (as opposed to all lower categories combined) by 84% since 1 did not fall within the CI (Table 4, Fig. 6). No order effect was detected in reaching a higher SUS, as the CI had a wide range and it encompassed 1. In terms of the test location–test order interaction (using the performance outdoors location and second test as reference), no significant effects were found (Table 4).

## Discussion

Our main aim in this study was to examine the effects of temperature, humidity, age, sex and neutering status on the olfactory performance of dogs tested outdoors in the NDT and to compare dogs’ olfactory performance in outdoor and indoor environments.

We found no evidence of a temperature effect in the NDT within the chosen temperature range (0–25 °C), which is consistent with the results of previous studies^20,21^. It is possible that the target scent used in this study had such an intensive fishy smell that the dogs had no problem finding it even at temperatures close to 0 °C, when lower amounts of odour may dissipate from the samples^22^. We chose 25 °C as the upper limit in our study because we expected that within this range, the dogs would be able to perform this simple olfactory task without the burden of intense, high-frequency panting^14^. Dogs were allowed to drink between the trials to rehydrate, which is known to help maintain nasal mucosal surface hydration in the olfactory epithelium^24^ and contribute to efficient odour retention. This may also explain why, contrary to some previous results^25–27^, humidity did not affect olfactory performance in our study.

We found that young adult dogs (2–3 years old) performed the best, while younger and especially older dogs were less successful, which is in contrast to some previous findings showing no effect of age in olfactory tasks on smaller samples^33–36^. It should be noted that our age groups may not perfectly align with all breeds, but correspond with the general lifespan status of the dogs^50^. The declining olfactory performance in our senior and geriatric dog groups may be explained by the known physiological changes occurring in aged dogs’ olfactory tracts^30,31^. However, socio-cognitive factors, such as concentration and persistence, also need to be considered. It is possible that neither young nor old dogs could concentrate long enough on this relatively long task, and/or they tended to lose their motivation sooner than young adults.

Due to the progressively more difficult nature of the task, it cannot be ruled out that, in addition to the less perceptible odour, other factors, such as fatigue, satiety, loss of motivation, or a combination of these contributed to the poorer performance at the more difficult levels.

We did not find a significant effect of sex and neutering status on either of the two metrics, similar to some earlier studies^33–35,38^. However, Abdel Fattah and Abdel-Hamid^39^, while testing more than 100 detection dogs, found that males and intact dogs performed better in olfactory search tasks than females and neutered dogs respectively. This may be explained by the difference in the samples in the two studies, trained German shepherd narcotic detection dogs versus untrained family dogs of various breeds. In the case of detection dogs, both the selection and the training are based on dogs’ ‘toy-drive’, as they are mostly motivated and rewarded by a ball, which requires a rather specific temperament and/or personality. In contrast, the family dogs in our test were not selected for this purpose and they were motivated by food, which motivation could be more easily extinguished than the ball motivation. As toy-drive can be assumed to play a crucial role in detection work (e.g.^16^), it is possible that intact male detection dogs are more motivated for the ball game than the other categories and this results in different performances. It would be particularly important to clarify this aspect because intact males are more commonly used for detection work than neutered males or females^41,43^. Working with intact males may be a personal preference, as suggested by Jamieson et al.^15^, or possibly just avoiding special effects of neutering on dog behaviour, particularly on the shyness-boldness personality scale (reviewed in^55^), that may affect working performance.

In line with our expectations and some previous studies^14,28^, we found a significant location effect; dogs tested indoors performed better in the NDT. This suggests that despite the relatively undisturbed test environment, dogs tested outdoors were more likely to be exposed to distracting visual and/or olfactory stimuli^18^, while an indoor laboratory setting may have created more standard conditions and better controlled for some environmental factors (as suggested by Horowitz and Franks^56^). In contrast to our findings, in a different setting Jezierski et al.^5^ found no difference in the olfactory performance of narcotic detection dogs indoors and outdoors. Still, they noted that in an outdoor location, the dog may easily be guided to the location of the scent source due to a more evenly distributed odour plume, while in an indoor location, the odour plume may fill the air space, which makes the tracking of the scent to the source difficult. The effect of test location was examined in a different context by Pongrácz et al.^57^ in a two-way-choice test, where dogs had to find the hidden food relying on human pointing signals. They found no difference between dogs’ outdoor and indoor performance, although the visual distraction outdoors may have been the same as in our test (same test locations). As the Pongrácz et al.^57^ test was based on using visual cues, we can assume that in our case, the major cause of disturbance in the outdoor environment was some odour stimuli.

The lack of significant order effect in the case of the repeatedly tested dogs suggests no considerable within-test learning effect, which is in line with the findings of Polgár et al.^18^ on a much smaller sample. This supports the relative temporal consistency of the NDT over time, which further validates this relatively simple test that requires no training. It should be noted though, that the lack of order effect may be due to the relatively long period of time between the test and the retest.

A possible limitation of our study is that its findings can mainly be interpreted on family dogs, and provide less reliable information regarding specially selected and trained detection dogs. At the same time, our analysis is not burdened by the disadvantages that occur during the testing of detection dogs, when it is difficult to draw conclusions about, for example, the effect of gender or age, since somewhat different training methods and test procedures inevitably hinder the collection of relevant sample sizes and thus a valid comparability.

The use of age categories instead of age as a continuous variable can also be seen as a potential limitation. Indeed, discarding small differences may reduce sensitivity. However, as we used seven age categories, each but the last (geriatric) category covering a range of 1–3 years only, rendering age as a semi-continuous variable, we do not think that the use of age categories seriously reduced sensitivity in the present study, even if dogs with a small age difference may in some cases have been put into different age categories. In contrast, using age categories may increase analysis robustness, also by downplaying variation which is assumably less relevant (for instance, the difference between the dog age 11 vs 12 years old may arguably be seen as less relevant than between 1 vs 2 years old). Whereas breed differences in aging make it impossible to link the onset of olfactory decline to a specific age in this study, the use of well-established, fine-grained age categories (e.g., see^49,50^) make our findings more comparable to previous works.No single study can answer all relevant questions relating to a complex performance in an olfactory task by analysing the data of one experiment. In this paper, we have focused on the examination of some environmental and demographic factors that are generally linked to the olfactory performance of dogs and voluntarily neglected some other potential influencing factors, such as breed effects or personality. Due to the unusually large sample size, the balanced samples, and the applied Bayesian statistical methods, these factors are unlikely to have significantly influenced the presented results.

It is important to take into account that in previous studies assessing the olfactory abilities of dogs, due to the small sample size, the effect of only a few potential influencing factors was always analyzed, so a significant effect was more likely to emerge. In the present study, despite the exceptionally large sample size, most factors did not show any or very robust effects, which suggests that even in such a simple behavioral test, an extremely large number of factors (and their interactions) can influence individual performance to some extent. This is a particularly instructive result if, for example, we want to assess the genetic predispositions behind this complex behavioural phenotype (success in an olfactory task).

## Conclusion

In sum, considering two types of success metrics, we demonstrated on a very large and diverse sample of family dogs that temperature (within the 0–25 °C range), humidity (within 18–90%), sex and neutering status did not significantly affect olfactory performance in the NDT. Age, however, did have an effect; young adults (2–3 years) were more successful compared to both younger (< 2 years) and older (> 6 years) dogs. A remarkable location effect was also revealed; dogs’ olfactory performance was better indoors compared to outdoors, possibly due to less distracting olfactory stimuli.

In conclusion, we demonstrated that family dogs’ olfactory performance is better in an indoor environment, however, they can be tested outdoors within 0–25 °C without the influencing effects of temperature and humidity. The lack of sex and neutering effect should be confirmed on a detection dog sample, so that the results can be generalised for applied purposes.

### Supplementary Information

Supplementary Information.

## Data Availability

All data are available as supplementary information.
